# Enhancing handicraft intangible cultural heritage learning through immersive VR serious games: a multimodal study on natural interaction effectiveness

**DOI:** 10.3389/fpsyg.2026.1880795

**Published:** 2026-07-16

**Authors:** Yupeng Jiang, Bingchen Gou

**Affiliations:** 1 School of Mechanical Engineering, Northwestern Polytechnical University, Xi'an, China; 2 Key Laboratory of Ministry of Industrial Design and Ergonomics, Ministry of Industry and Information Technology, Northwestern Polytechnical University, Xi'an, China; 3 Shaanxi Engineering Laboratory for Industrial Design, Northwestern Polytechnical University, Xi'an, China

**Keywords:** electrodermal activity, eye-tracking, handicraft intangible cultural heritage, natural interaction, serious game, virtual reality

## Abstract

**Introduction:**

With the integration of natural interaction technologies such as gesture recognition and eye tracking into virtual reality (VR) systems, new possibilities have emerged for VR-based education of intangible cultural heritage (ICH) craftsmanship. Using Bai ethnic tie-dyeing as a case, this study developed an immersive virtual reality serious game (IVR-SG) grounded in Self-Determination Theory (SDT) to explore the differences between natural and controller-based interaction in VR learning of handicraft ICH.

**Methods:**

A total of 68 participants were randomly assigned to a natural interaction group or a controller interaction group. Learning processes were assessed through eye-tracking, electrodermal activity (EDA) and questionnaires.

**Results:**

The results revealed three key findings. First, participants using natural interaction demonstrated higher attentional focus during craft process simulation and faster command selection, but exhibited higher cognitive loads in precision tasks. Second, the natural interaction group reported significantly higher flow experience and self-efficacy. Third, the natural interaction group achieved significantly higher post-test knowledge scores, while both groups achieved comparable in-game procedural performance. Furthermore, regression analysis indicated that prior knowledge, flow experience, self-efficacy, and blink rates were positively correlated with knowledge acquisition.

**Discussion:**

These findings highlight the differential impacts of interaction modalities in VR-based handicraft ICH education and provide design references for optimizing natural interaction in VR serious games for ICH.

## 1 Introduction

Traditional handicraft intangible cultural heritage (ICH) includes the skills of manual labor and related products, forming an important part of ICH (Yang et al., 2018). However, the transmission of handicraft ICH faces considerable challenges. Traditional handicraft practice demands substantial skill acquisition yet offers relatively modest economic returns, resulting in diminished engagement among younger generations. Moreover, the inherently localized character of such heritage constrains conventional apprenticeship models that rely on oral instruction and in-person demonstration, thereby limiting their temporal flexibility, spatial accessibility, and pedagogical efficiency (Carrozzino et al., 2011). Many handicraft ICHs are at risk of being lost, thus necessitating innovative strategies and methods for their dissemination.

Virtual Reality (VR) technology, as an emerging digital technology, has been actively applied to the learning and experiencing of ICH, such as martial arts, theater, and folk activities (!!! INVALID CITATION !!! Makransky and Mayer, 2022; Selmanovic et al., 2018; Xiao and Guo, 2021; Makransky and Mayer, 2022; Selmanovic et al., 2018; Xiao and Guo, 2021). The research findings indicate that VR technology, through its interactive three-dimensional dynamic visuals and system simulation of physical behaviors, enables users to complete remote exploration and learning tasks effectively. Furthermore, its integration with serious gaming to create virtual reality serious games (VR-SGs) provides users with enhanced learning motivation and self-efficacy (D'Amico et al., 2023). Notably, participants in immersive virtual reality (IVR)-based SGs demonstrate significantly higher levels of knowledge retention and physiological arousal compared to those engaging with traditional PC-based VR-SGs (Chittaro and Buttussi, 2015). However, while these studies have confirmed the potential of IVR-SG in ICH education, existing research has predominantly focused on performance-based or ritual ICH, with limited attention to handicraft ICH, which emphasizes the execution of sequential manual operations to process materials. Meanwhile, with the continuous advancement of VR hardware, devices such as the HTC Vive Focus 3, Apple Vision Pro, and Meta Quest have achieved breakthroughs in natural interaction modalities through technologies like eye-tracking and gesture recognition. VR interactivity shapes key cognitive and affective processes (including attention allocation, embodiment, and intrinsic motivation) that can either support or impede learning outcomes (Petersen et al., 2022). Yet, how these emerging natural interaction methods differ from traditional controller-based approaches in terms of cognitive engagement, attentional allocation, and learning experience within skill-based handicraft ICH VR training remains an open question lacking empirical evidence.

To investigate the differential impacts of VR interaction modalities on handicraft ICH learning, a well-designed IVR-SG platform is essential as the research foundation. Effective digital games require the joint creation of games and psychological theories (Xi and Hamari, 2019). A systematic review of 118 theories in gamification and serious-game research identified SDT as by far the most frequently applied theoretical framework for game design, followed by scaffolding theory, the ARCS motivation model, and Social Cognitive Theory, among others (Krath et al., 2021). However, these frameworks differ substantially in the scope and specificity of design guidance they afford. Unlike scaffolding theory, which addresses only the skill-acquisition support dimension, or the ARCS model, whose Confidence category conflates autonomy and competence, SDT functions as a generative design theory: its three basic needs—autonomy, competence, and relatedness—specify which environmental conditions foster intrinsic motivation (Sailer et al., 2017) and translate directly into actionable game mechanics (Peters et al., 2018). This is particularly relevant to handicraft ICH learning, where sustained engagement and active skill practice are essential for mastering complex manual procedures. Moreover, SDT bears directly on the interaction-modality question that is central to this study. Within SDT, competence is defined as the need to feel effective in interacting with one's environment (Ryan and Deci, 2017); the manner of VR interaction—natural gesture-and-gaze input vs. handheld controller—therefore has direct theoretical implications for need satisfaction. Drawing on the SDT-based METUX model (Peters et al., 2018), which distinguishes the interface sphere (interaction with controls) from the task sphere (the learning activity itself), the present study positions interaction modality as an interface-sphere variable and the learning phases as task-sphere variables, thereby yielding a theoretical basis for expecting phase-dependent rather than uniform interaction effects. SDT thus serves a dual role in this study: at the design level, it specifies a need-supportive platform held constant across both experimental conditions; at the analytic level, through METUX, it provides the mechanism by which interaction-modality differences are predicted and interpreted.

Specifically, employing the Bai ethnic tie-dye craft, an ICH practice from Yunnan, China, as an empirical case, this study develops an SDT-guided IVR-SG to systematically compare the effects of natural interaction and controller-based interaction on learning effectiveness. A multimodal assessment framework combining physiological monitoring (eye-tracking, galvanic skin response) and psychological evaluation (self-efficacy scales, flow state inventories) was implemented to quantitatively compare user experience across interaction paradigms. The learning outcomes are evaluated using a pre-test of previous knowledge and a post-test on knowledge acquisition. Our research questions (RQs) are as follows:

RQ1. How do natural and controller-based interaction differentially affect cognitive engagement across learning phases in an IVR-SG for handicraft ICH?
RQ2. What are the effects of interaction modality on learning experience and outcomes in an IVR-SG for handicraft ICH?
RQ3. To what extent do cognitive engagement and learning experience predict knowledge acquisition in an IVR-SG for handicraft ICH?

## 2 Literature review

### 2.1 ICH education and IVR-SG

VR technology offers promising solutions (Cui, 2023). Recent work indicates that SGs are increasingly adopted for knowledge and skill training (Solinska-Nowak et al., 2018). Designed primarily for education, SGs shift learning from didactic lecture delivery toward more engaging, emotionally rich experiences. Combining SG with IVR technology creates IVR-SG, which enhances student learning performance compared to traditional methods (Fujimi and Fujimura, 2020). IVR-SG has become a popular tool for the experience and teaching of ICH. For example, Liu et al. (2022) designed an interactive IVR system called Hua'er and Youth (HY), which integrates three methods: virtual avatars, participatory performance, and game-based knowledge acquisition. This system helps users experience and understand the traditional Chinese oral performance known as Hua'er. Lu et al. (2023) designed an IVR-SG based on the features of three types of pictograph construction methods to help users gain a deeper understanding of the structural characteristics and meaning of the Dongba intangible script. Rodil et al. (2022) designed an IVR-SG that leverages embodied learning to convey the cultural and chemical aspects of Umqombothi, a traditional South African beer, through a realistic township brewing environment and an abstract “Microverse” space.

However, while some studies have confirmed the feasibility of incorporating IVR-SG into ICH education, research on virtual reality programs for handicraft ICH is limited, and most educational intervention designs of IVR-SG lack a theoretical foundation. To this end, it is advisable to integrate relevant theories into IVR-SG to enhance the effectiveness of learning and training. Among the literature, SDT has piqued our interest.

### 2.2 Self-determination theory

Self-determination theory (SDT), developed by Deci and Ryan (2013) and refined through subsequent research, is a macro-theory of human motivation positing three innate basic psychological needs: autonomy (the need to experience volition in one's actions), competence (the need to feel effective in interacting with the environment), and relatedness (the need to feel connected to others). When these needs are adequately satisfied, individuals exhibit enhanced intrinsic motivation, effective self-regulation, and greater wellbeing (Ryan and Deci, 2000). While alternative frameworks such as scaffolding theory, the ARCS motivation model, and Social Cognitive Theory have yielded positive results in heritage and game-based learning (Hao and Lee, 2021; Li et al., 2024; Ye et al., 2021), each addresses only a partial dimension of the present design problem: scaffolding theory does not account for learner autonomy or social relatedness (Wood et al., 1976), the ARCS model conflates autonomy and competence within its Confidence category (Baah et al., 2023), and Social Cognitive Theory does not map psychological needs to game mechanics. SDT provides a more comprehensive motivational architecture by subsuming scaffolding within its competence dimension (Chen and Law, 2016), accommodating self-efficacy as a measurable outcome aligned with the competence need, and simultaneously specifying the conditions for autonomy and relatedness satisfaction. This integrative capacity has supported its application across diverse game-based learning contexts: Buil et al. (2019) demonstrated that the satisfaction of all three basic needs positively predicted cognitive, emotional, and behavioral engagement in a business simulation game; Li et al. (2022) showed that SDT-guided design of augmented reality serious games enhanced children's learning motivation and social interaction; and Tan et al. (2023) validated the feasibility of an SDT-based digital board game in fostering autonomous rehearsal motivation among medical students learning anatomy.

Beyond its role as a design framework, SDT is directly relevant to the interaction-modality question central to this study. SDT defines competence as perceived effectiveness in interacting with one's environment (Ryan and Deci, 2017); the manner in which learners interact with a VR system therefore has direct implications for whether they experience competence satisfaction or frustration. Empirical evidence supports this connection, with studies demonstrating that VR interaction modality influences user experience through competence and autonomy satisfaction (Reer et al., 2022; Yang and Li, 2025). The METUX model (Peters et al., 2018) offers a more fine-grained account by distinguishing the interface sphere, concerning immediate interaction with controls, from the task sphere, concerning the learning activity itself. This distinction yields a specific prediction: because a single interaction modality may satisfy competence at the interface sphere while frustrating it at the task sphere, or vice versa, its effect on cognitive engagement should be phase-dependent rather than uniform. Moreover, both competence and autonomy satisfaction have been established as predictors of flow experience (Kowal and Fortier, 1999; Valenzuela et al., 2018), indicating that interaction modality may influence learning through multiple need-satisfaction pathways beyond the competence dimension alone. On this theoretical basis, we developed the Tie-dye IVR-SG according to SDT's three fundamental needs, providing a learning environment in which the effects of different interaction modalities on learner engagement and outcomes can be systematically examined.

### 2.3 Natural VR interaction method

Traditional immersive VR interaction relies primarily on handheld controllers. As VR hardware has matured, however, interaction modalities have shifted toward more natural and intuitive paradigms. Gesture interaction, based on non-invasive sensors for tracking body movements and gestures, can enable non-contact operation of digital content or remote devices (Vuletic et al., 2019). Eye-tracking technology uses infrared light sources to illuminate the eyes and cameras to capture reflected light points and pupil positions, thereby calculating the user's gaze point and tracking the dynamic changes of their line of sight in real time. Eye-tracking also helps to enhance the interactive experience in VR (Adhanom et al., 2023). In devices such as HTC VIVE Focus Vision, Apple Vision Pro, and HoloLens 2, users can select interface elements (e.g., menu items or icons) through gaze-based targeting.

Recent studies comparing hand tracking and controller-based interaction in VR have revealed a trade-off between naturalness and control fidelity. Controllers consistently yield faster task completion, higher precision, and lower cognitive load than hand tracking (Hameed et al., 2021), while hand tracking enhances embodiment but suffers from lower learnability in complex tasks (Steed and Lai, 2025). Moreover, prolonged mid-air gesture interaction can induce arm fatigue, compounded by tracking inaccuracy that forces users to repeat gestures (Rantamaa et al., 2023). These concerns are particularly relevant to handicraft ICH learning, which requires sustained manual operations, yet existing comparisons have been conducted primarily in general-purpose VR tasks rather than in skill-based educational contexts where interaction itself constitutes part of the learning objective. However, although multimodal natural interaction techniques integrating gestures and eye-tracking have demonstrated advantages over traditional controller-based approaches in numerous VR studies, it remains unclear whether such novel interaction paradigms might impose substantial learning burdens on users within IVR-SG designed for ICH craftsmanship preservation.

### 2.4 Flow experience and self-efficacy

Flow, originally conceptualized by Csikszentmihalyi (1975), refers to an optimal psychological state in which individuals become fully absorbed in a goal-directed activity. Nakamura and Csikszentmihalyi (2014) specified nine dimensions of flow, among which three function as antecedents directly shaped by how learners interact with a virtual environment: challenge-skill balance, clear goals with immediate feedback, and sense of control. Empirical evidence consistently links flow with task persistence and knowledge retention in VR-based skill training (Kiili, 2005). Among available instruments, the Flow Short Scale (FSS) condenses the nine dimensions into two validated factors (Engeser and Rheinberg, 2008): fluency of performance, which indexes smooth, automatic action execution, and absorption by activity, which indexes the depth of attentional engagement. This two-dimensional structure is well-suited for comparing interaction modalities, as fluency is theoretically sensitive to how directly user input translates into virtual action, whereas absorption reflects the extent to which interaction overhead competes with content-focused attention.

Self-efficacy refers to an individual's belief in their capability to organize and execute the actions required to attain a given performance, a concept grounded in social cognitive theory (Bandura, 1977). Bandura identified four sources of efficacy beliefs, of which mastery experience and physiological and affective states are most directly implicated by embodied VR interaction, since hand-tracking approximates the kinematics of authentic craft practice and lower interaction friction elicits more positive affective states during task execution. The Cognitive Affective Model of Immersive Learning (CAMIL) further positions self-efficacy as a key mediator between VR affordances and learning outcomes (Makransky et al., 2021), a pathway empirically supported in IVR-based skill studies (Tai et al., 2022). Self-efficacy also aligns conceptually with the competence need that anchors the SDT-guided game design described in Section 2.2. To capture the construct in a manner matching the dual evaluation of subjective experience and objective knowledge in the present study, we adopt the Motivated Strategies for Learning Questionnaire-Self-Efficacy (MSLQ-SE), whose two facets, respectively, reflect confidence in mastering craft content and confidence on the associated assessment: specific academic learning self-efficacy (SAL-SE) and specific academic exam self-efficacy (SAE-SE; Nielsen et al., 2017).

### 2.5 Cognitive load and physiological measurement in VR learning

Cognitive Load Theory (CLT), developed by Sweller (1988), provides a theoretical framework for understanding how instructional design affects the processing demands placed on learners' working memory. CLT identifies three types of cognitive load: intrinsic load, inherent to the complexity of the learning material; extraneous load, imposed by the manner in which information is presented; and germane load, devoted to schema construction and meaningful learning (Sweller, 2011). In VR learning environments, extraneous cognitive load is of particular concern because it can be induced by both the interaction technique and the complexity of the virtual environment (Makransky et al., 2019). Grounded in CLT, the present study employs two complementary physiological channels to objectively capture cognitive load fluctuations during the learning process: eye-tracking blink rate, which indexes the perceptual-attentional demands of information processing via the oculomotor system, and electrodermal activity (EDA), which indexes the autonomic arousal elicited by mental effort via the sympathetic nervous system (Ayres et al., 2021; Hogervorst et al., 2014).

Eye-tracking captures fixations, saccades, and blinks as indicators of visual attention and cognitive state (Rosch and Vogel-Walcutt, 2013), and has been increasingly adopted in IVR educational research. For instance, Dubovi (2022) used fixation duration and pupil diameter to show how visually similar drug packaging elevated cognitive load among nursing students during VR medication training. Beyond fixation-based metrics, blink rate has been employed as an indicator of cognitive load in applied settings: Wilson (2002a) found that pilots' blink rates decreased during the most visually demanding segments of flight, such as takeoffs and landings, reflecting the suppression of blinks to maintain continuous visual information intake under elevated cognitive demand; and Page et al. (2024) reported that blink rate decreased systematically with increasing cognitive load during a complex real-world multitasking scenario, confirming its sensitivity as a continuous indicator of cognitive demand.

EDA (also Galvanic Skin Response, GSR) reflects arousal-related changes in skin conductance and varies systematically with alertness, mental workload, and task difficulty (Altintop et al., 2020), making it well-suited for capturing the autonomic arousal dimension of cognitive load under the dynamic conditions of virtual environments (Hogervorst et al., 2014). Using mean skin conductance level (SCL), Armougum et al. (2019) found that novice travelers exhibited higher EDA-indexed cognitive load than experienced travelers in a VR navigation task, whereas Liberman and Dubovi (2023) reported that the frequency of skin conductance responses (SCRs) was sensitive to cognitive load differences between verbal-narration and on-screen-text conditions during VR learning. Together, these studies establish EDA as a viable index of cognitive load in immersive contexts, with phasic SCR measures such as peak frequency showing particular sensitivity to task-related cognitive demands.

## 3 The tie-dye IVR-SG

Bai ethnic tie-dye is a traditional resist-dyeing craft practiced in the Dali region of Yunnan, China. It produces distinctive patterns on fabric by tying, stitching, or folding the cloth to prevent dye from reaching selected areas. Mastering this craft goes beyond cultural knowledge. It is achieved through embodied learning, which involves active hand operations on the fabric and repeated practice of the craft's sequential steps. Although conventional museum exhibitions effectively preserve and display finished artifacts, they remain limited in conveying the procedural and action-based dimensions of craft expertise. To address this gap, the present study situates the IVR-SG within a digital museum environment. The structured spatial layout of the virtual museum supports the systematic delivery of cultural and material knowledge, while the serious game allows learners to perform simulated craft operations. Together, these features extend the museum experience from passive appreciation to active skill acquisition.

The design of the Tie-dye IVR-SG is primarily based on the SDT. The game includes five phases: Tie-dye Digital Museum, Tie-dye Pattern Design, Tie-dye Process Simulation, Tie-dye Virtual Gallery, and Unlock New Resources. To ensure a transparent correspondence between theory and design, the three basic psychological needs of SDT were systematically operationalized through specific design features embedded in each game phase. Table 1 presents this need-to-design mapping, illustrating how autonomy, competence, and relatedness are addressed through concrete game mechanics and the theoretical mechanisms through which each feature is expected to support the corresponding psychological need. Critically, all SDT-based design features listed in Table 1 were held constant across both the natural interaction and motion controller conditions; the two groups experienced identical game content, phase structure, and scaffolding support. The only between-group difference was the interaction modality itself, which constitutes the independent variable of this study.

**Table 1:** Mapping of SDT basic psychological needs to game design features in the Tie-dye IVR-SG.

SDT need	Game phase	Design feature	Theoretical mechanism
Autonomy	3.2 Pattern Design	Free selection of motifs and composition styles; personalized creation	Supporting self-endorsed creative expression enhances perceived autonomy
Competence	3.3 Process Simulation	Progressive scaffolding; action prompts; error feedback with explanation; action rating	Optimal challenge with informational feedback supports effectance motivation
	3.5 Unlock Resources	Points reward system; progressive unlocking of new motifs and models	Tangible progress indicators serve as informational (not controlling) competence feedback
Relatedness	3.4 Virtual Gallery	Artwork sharing; browsing others' creations; bookmarking; collaboration invitations; artwork transactions	Social interaction and community engagement foster sense of connectedness

### 3.1 Tie-dye Digital Museum

**Figure 1:**
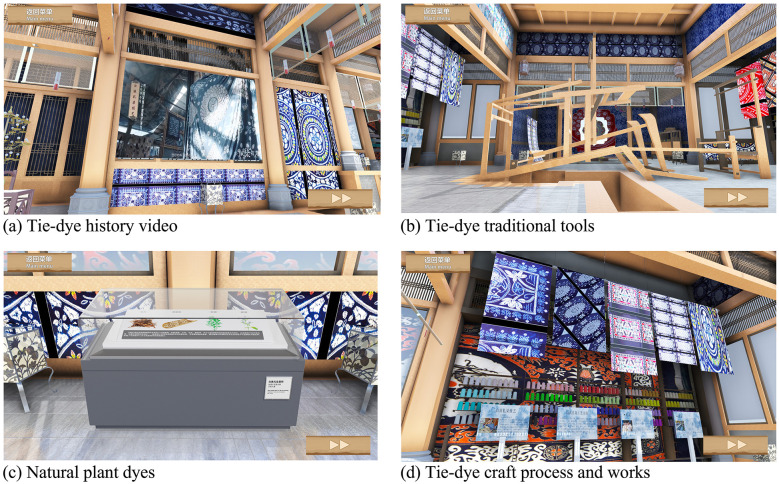
Functional areas of the Tie-dye Digital Museum. **(a)** Multimedia screens presenting tie-dye history and classic pattern displays. **(b)** Traditional tie-dye tools and wooden looms exhibited alongside finished works. **(c)** Glass display cabinet containing natural plant dye specimens with descriptive labels. **(d)** Gallery of tie-dye craft processes and finished works.

When learners first enter the Tie-dye IVR-SG, they can watch multimedia videos on the large screens of the Digital Tie-dye Museum to understand the cultural background, aesthetic features, and folklore significance of the Bai ethnic group's intangible cultural heritage tie-dye techniques. Learners can also see specimens of natural plants used as dyes, such as indigo, isatis root, and mugwort, in the display cabinets of the museum. The museum displays models of traditional tools accompanied by operation diagrams to provide learners with a basic understanding of the tie-dyeing process, and there are also many classic tie-dye works for people to appreciate. The virtual environment of the museum is shown in Figure 1.

### 3.2 Tie-dye Pattern Design

**Figure 2:**
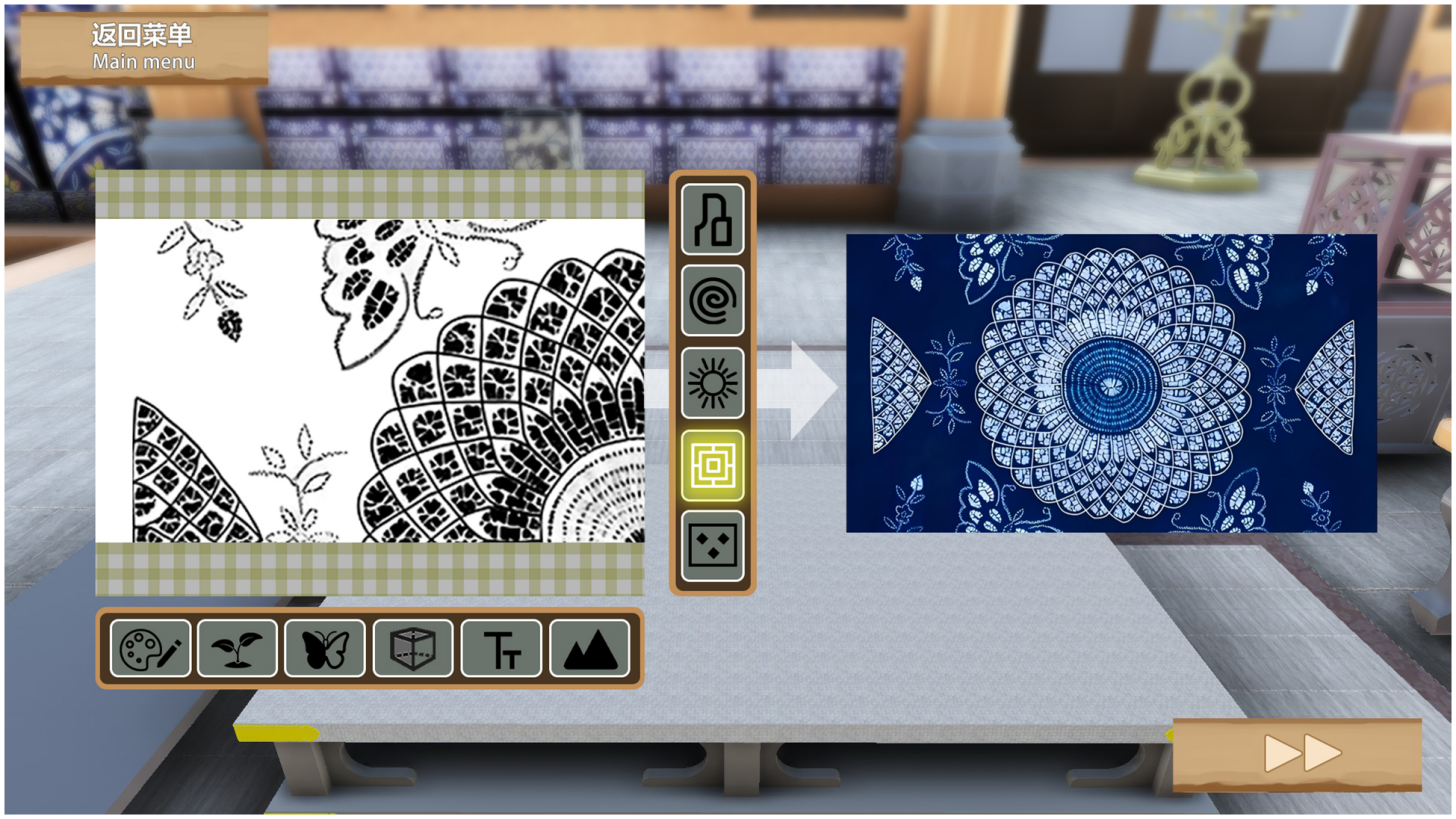
Game view of the pattern design phase. **(Left)** the drawing canvas where learners assemble selected motifs into base patterns. Bottom: the tool panel providing six motif categories (e.g., floral, botanical, and geometric). **(Center)** composition style panel with seven configuration options. **(Right)** the pattern preview template rendering the real-time dyeing effect on indigo-dyed fabric.

Game creatability represents a game's ability to give players the power to create. The IVR-SG enables learners to design personalized tie-dye patterns through traditional motifs or innovative concepts, fulfilling their autonomy needs as defined by SDT. As shown in Figure 2, the virtual reality space at this phase comprises three primary components: the drawing canvas, the tool panel, and the pattern preview template. The tool panel provides traditional Bai ethnic tie-dye motifs and composition configurations for selection, including six motif categories and seven traditional composition styles. Learners assemble selected motifs on the canvas to create base patterns, then finalize designs through composition style configuration. The VR preview template enables real-time visualization of dyeing effects on fabric through photorealistic material rendering, allowing iterative design refinement.

### 3.3 Tie-dye Process Simulation

**Figure 3:**
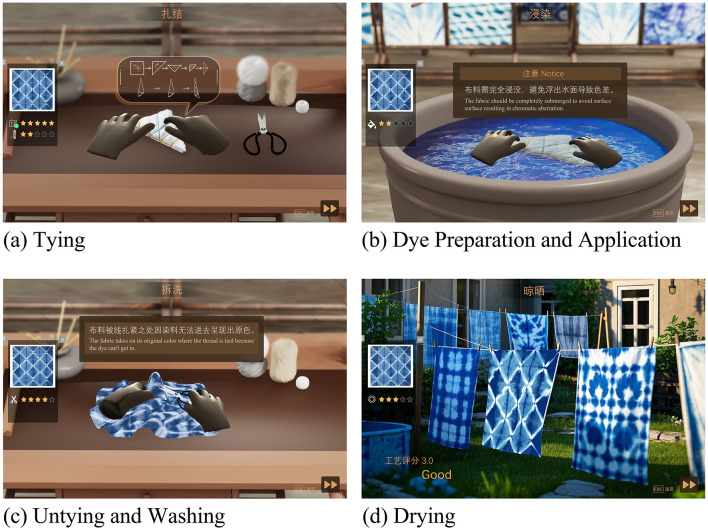
Sample of virtual environment for Tie-dye process. **(a)** Tying: folding and binding fabric with thread, guided by an action prompt displaying the folding diagram. **(b)** Dyeing: submerging tied fabric into a dye basin, with an instructional notice explaining the correct technique. **(c)** Untying and washing: rinsing the dyed fabric, accompanied by explanatory feedback on the resist-dyeing principle. **(d)** Drying: finished fabrics displayed on clotheslines with a craftsmanship rating score.

This phase comprises four craft procedures: tying, dyeing, washing, and drying (see Figure 3). By interacting with virtual objects to complete each task, learners progressively acquire the corresponding tie-dye techniques. Chen and Law (2016) argue that scaffolding in games can not only help in situations where players are frustrated with learning but can also stimulate creative problem-solving, enhancing players' competence needs as defined by SDT. Thus, we incorporated active scaffolding and passive scaffolding into the IVR-SG design.

When users engage in the tie-dye process within an immersive virtual reality environment, the main working area for operations is in the center of their vision. Action prompts and dynamic visual demonstrations appear in the upper visual area to guide interactions. Upon operational errors, the system provides auditory error notifications with explanatory feedback, helping users correct their actions and understand the underlying principles. Additionally, during the tie-dye process, the left side of their vision displays the progress of completion. After each operation, there is an action rating, providing feedback that can enhance users' self-efficacy and also meet their competence needs (Xi and Hamari, 2019).

### 3.4 Tie-dye Virtual Gallery and Unlock New Resources

In the Tie-dye IVR-SG, users can apply their completed tie-dye fabrics to items such as canvas bags, clothing, and dolls, thereby owning their own personalized merchandise. Users can upload their favorite creations to an online gallery. In addition, strengthening interactions among different players can also meet the relatedness needs of players proposed in SDT Kuem et al., (2020). Therefore, the Tie-dye IVR-SG allows learners to browse others' tie-dye artworks in the virtual gallery, bookmark preferred creations, contact creators for artwork transactions, and send collaboration invitations to jointly complete projects.

The mode of unlocking new features in the game can enhance the fulfillment of the need for competence (Hamari, 2017). The virtual reality tie-dye platform has established a points reward system, where learners can earn points by completing tasks and trading works. These points can be used to unlock more tie-dye motifs and tie-dye-related models in the store, enabling them to create more diverse tie-dye works.

## 4 Methods

This study conducted a randomized controlled trial with two parallel groups to examine the impact of VR interaction modalities on the learning of handicraft ICH. Participants were randomly assigned to either the experimental group (Natural interaction) or the control group (Motion controller interaction). Learning outcomes were assessed through a multimodal approach, incorporating subjective self-reports and objective physiological measurements to capture both cognitive processes and performance.

### 4.1 Participants

Prior to data collection, an a priori power analysis was conducted using G^*^Power 3.1 (Faul et al., 2009) to determine the minimum sample size for the hierarchical multiple regression analysis, which included six predictors. Based on the large effect sizes reported in meta-analyses of immersive VR educational interventions (Villena-Taranilla et al., 2022), a large anticipated effect size (Cohen's *f* ^2^ = 0.35) was specified, with α = 0.05 and power = 0.80, yielding a minimum requirement of 46 participants. Accordingly, this study recruited 72 students from a large 4-year university in the Northwest region of China. Recruitment materials comprised email advertisements and posters on bulletin boards in academic buildings, and students were reminded that participation in the study was strictly voluntary. Traditional Bai ethnic tie-dye practice exhibits a gendered division of labor, where women predominantly tie and both sexes dye. However, because our study aimed to evaluate VR interaction methods rather than replicate real-world demographics, we recruited a gender-balanced sample. This approach controls for physiological confounds and ensures the generalizability of our findings across learners. In addition to gender balancing, participants were screened against the following exclusion criteria: abnormal stereo vision, strabismus, retinal disease, visual field defects or other eye diseases, and limitations in body movement and balance. Prior VR experience was also assessed during the screening phase; all 68 participants reported no previous experience with head-mounted VR devices, ensuring that between-group comparisons were not confounded by differential technological familiarity. Four participants were excluded due to eye-tracking calibration errors, yielding a final sample of 68 participants (32 females, *M*_age_ = 22.2, *SD*_age_ = 3.0; 36 males, *M*_age_ = 23.9, and *SD*_age_ = 3.3). Participants were randomly assigned to one of two groups: the Motion controller interaction or the Natural interaction, with 34 participants in each group. Written informed consent was obtained from all participants. The study was reviewed and approved by the Biomedical Ethics Committee of Northwestern Polytechnical University and was conducted in accordance with the guidelines of the Declaration of Helsinki.

### 4.2 Software and hardware

**Figure 4:**
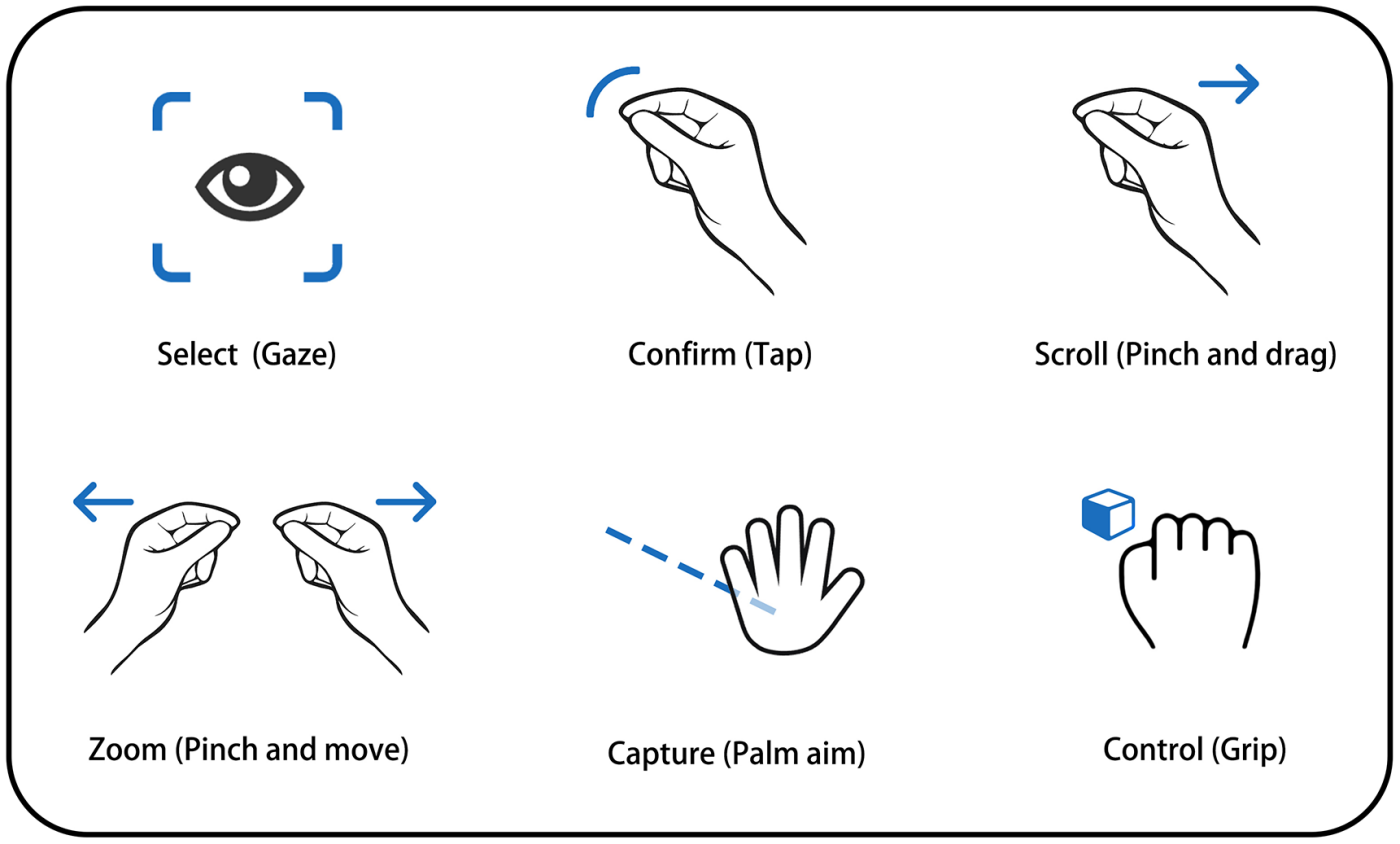
VR natural interaction postures.

We developed the Tie-dye IVR-SG using Unity (version 2022.3 LTS, Universal Render Pipeline). Frame rate was monitored via Unity's built-in Profiler and maintained above 90 FPS throughout all sessions, exceeding the 90 FPS baseline established as the industry standard for comfortable VR experiences (Wang et al., 2023). Unity's XR Interaction Toolkit provided ready-made components for implementing VR interactions. The open-source modeling software Blender (version 4.2) was used to create custom 3D models; the remaining models were obtained from the Unity Asset Store and other online repositories. Gaze- and gesture-based interaction commands were configured using the XR Gaze Interactor and VIVE Wave SDK (version 5.6.0). The VIVE Wave SDK provides a vision-based hand tracking engine that detects 26 skeletal joint points per hand via the head-mounted display (HMD)'s onboard cameras and supports six predefined gesture types. Prior to formal data collection, each participant completed a calibration session of 10 trials per required gesture to confirm stable recognition. The natural interaction condition in this study is operationalized as a coordinated multimodal input scheme comprising gaze and hand gesture channels (see Figure 4). Gaze input serves as the primary selection modality, while hand gestures fulfill a spectrum of manipulation functions, including action confirmation (Tap), content navigation (Pinch and drag), view scaling (Pinch and move), object acquisition (Palm aim), and tool manipulation (Grip). Within this framework, the two channels operate in a complementary division of labor consistent with the gaze-assisted manipulation paradigm increasingly adopted in commercial VR platforms (e.g., HTC VIVE Focus Vision, Apple Vision Pro, and Meta Quest). The controller-based condition, by contrast, relies on a single handheld device for both selection and manipulation.

**Figure 5:**
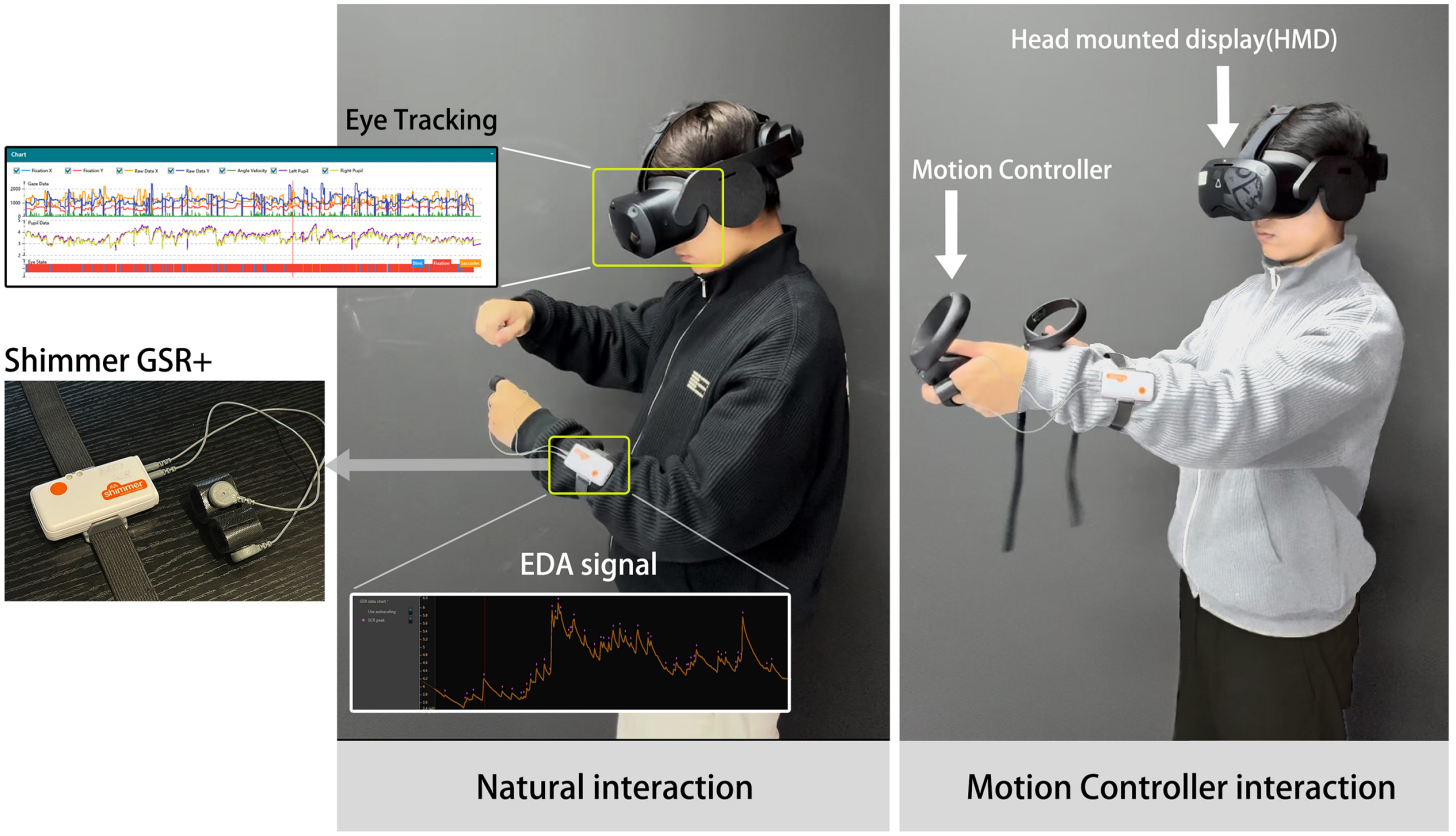
The scene of the participant in the experiment trial. **(Left)** Participant experience IVR-SG through Natural interaction; **(Right)** Participants experience IVR-SG through Motion controller interaction.

The VR device used in this study was the HTC Focus 3 (HTC Corporation Inc.), which features a monocular resolution of 2,448 × 2,448 and a 120° field of view, a refresh rate of 90 Hz, and it supports motion tracking without the need for a base station. It has a magnetically attached eye tracker, and the eye tracker has a data output frequency of up to 120 Hz and an accuracy range of 0.5°-1.1°. Raw data provided by the eye tracker includes the origin of the gaze, gaze vectors, eye openness, pupil diameter, and data validity. Raw data from the eye tracker was obtained using the HTC SRanipal SDK as CSV files and subsequently processed and analyzed using ErgoLab 3.0 software (Kingfar International Inc., Beijing, China). EDA was continuously recorded with a Shimmer3 GSR+ sensor (Shimmer Research, Dublin, Ireland). The device was secured to the participant's wrist, with two electrodes attached to the index and middle fingers of their non-dominant hand, as shown in Figure 5. The EDA data were collected and processed using Tobii Pro Lab software.

### 4.3 Procedure

**Figure 6:**
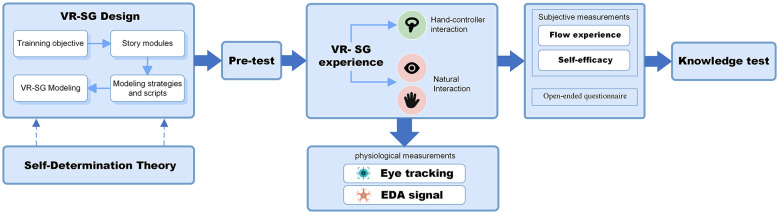
Experimental design and data collection procedure.

Figure 6 presents a flowchart illustrating the experimental procedure of this study. Prior to the VR experience, each participant completed a demographic questionnaire (sex, age, profession, level, and field of study) and a tie-dye skills and experience assessment. Participants in the experimental group achieved posture synchronization between the virtual hand and their real hand through the HMD's hand-tracking functionality, with eye-tracking facilitating rapid target selection (see Figure 5, Left). The control group manipulated the virtual hand in the virtual environment by moving and pressing motion controllers, where the virtual hand's movements were automatically adjusted to match the operational targets (see Figure 5, Right).

Before formally experiencing the VR-SG, each participant was required to wear a VR HMD and undergo eye-tracking calibration, followed by a 10-mins training session to familiarize themselves with the operations and adapt to the virtual environment. Due to the distinct hardware configurations required for the two interaction conditions, experimenters were necessarily aware of group allocation; to minimize potential bias, all participants received identical standardized instructions, and the experimenter's role was limited to equipment setup and safety monitoring throughout the sessions. Once both groups were prepared, they engaged in the VR-based simulation learning. The IVR-SG consisted of five phases, with participants taking a 5-mins rest after completing each phase. Throughout the learning activities, all participants received identical embedded learning support, as detailed in Section 3.3 and Table 1.

Following the learning session, participants completed the flow experience and self-efficacy questionnaires (administered post-session to preserve immersion continuity) and were invited to complete an open-ended questionnaire about their learning experience (see Section 4.4.3 and Appendix C). Finally, a post-knowledge test was conducted 1 week after the completion of the experience. The test was not completed immediately after the experience, because delayed tests are particularly useful when determining the persistence of learning outcome effects (Checa et al., 2023). All 68 participants completed the delayed post-test, with no attrition during the 1-week interval.

### 4.4 Data collection instruments

The multimodal assessment framework of this study integrates physiological, psychological, and knowledge-based measures. Table 2 summarizes the five constructs examined, along with their corresponding indicators, data types, and operational metrics.

**Table 2:** Detailed description and definition of study variables by the type of modality.

Construct	Variable	Data type	Metric
Cognitive load	Blink rate	Objective, continuous (eye-tracking)	Number of blinks per minute
	EDA peak frequency	Objective, continuous (electrodermal activity)	Number of phasic skin conductance response peaks per minute
Attentional engagement	Time to first fixation (TTFF)	Objective, continuous (eye-tracking)	Time elapsed from AOI onset to the first fixation on the AOI (ms)
	Average fixation duration (AFD)	Objective, continuous (eye-tracking)	Mean duration of all fixations within an AOI (ms)
	Fixation dwells	Objective, continuous (eye-tracking)	Total fixation duration on an AOI as a percentage of total fixation duration across all AOIs (%)
Flow experience	Flow Short Scale (FSS) score	Subjective, post-test (self-report)	Descriptive statistics (Mean, SD) were calculated for the Flow Short Scale.
Self-efficacy	MSLQ-SE score	Subjective, post-test (self-report)	Descriptive statistics (Mean, SD) were calculated for the MSLQ-SE.
Knowledge acquisition	Pre-test score	Objective, pre-intervention (knowledge test)	Knowledge level was calculated as the Mean (SD) total correct score.
	Post-test score	Objective, post-intervention (knowledge test)	Knowledge level was calculated as the Mean (SD) total correct score.

#### 4.4.1 Cognitive engagement

Eye-tracking. Eye movement metrics extracted from specific Areas of Interest (AOIs) offer detailed insights into participants' visual attention allocation and cognitive prioritization within the virtual environment. Therefore, prior to data collection, AOIs in this study were predefined based on the primary functional components of each game phase described in Section 3. For the Tie-dye Pattern Design phase, three AOIs were designated: the drawing canvas, tool panel, and pattern preview template (Figure 2). Similarly, for the Tie-dye Process Simulation phase, the three assigned AOIs comprised the virtual hands, fabrics, and auxiliary toolbar (Figure 3). This task-driven approach ensured that the AOIs captured the key areas where interaction method differences were most likely to influence visual attention.

Subsequently, we analyzed differences in eye-tracking data between the experimental and control groups within specific AOIs to investigate how distinct virtual reality interaction modalities influenced participants' learning in the tie-dyeing IVR-SG. Calculations were performed for the following metrics: average fixation duration (AFD in milliseconds), time to first fixation (TTFF), blink rate, and the total fixation duration per AOI expressed as a percentage of the total fixation duration across all AOIs. (Robus et al. 2020) found that during goal-directed tasks, longer AFD reflects greater difficulty in processing the attended visual content. Based on TTFF, it is possible to determine the order in which users visually prioritized AOIs within the interface to infer the relative salience of different AOIs to participants. Another important eye-tracker metric is the blinking rate. Higher task difficulty suppresses blink rate, reflecting increased cognitive load (Holland and Tarlow, 1975); conversely, higher blink rates indicate lower cognitive load during task performance. Fixation duration provides insights into higher-level viewing behavior. High absolute fixation duration is taken as an indicator of high information value. High relative fixation duration on specific AOIs points to essential and informative objects (Conati et al., 2020).

Electrodermal activity. SCR activity corresponds to the combined interactions of several neural pathways of the human brain (Critchley et al., 2000). Nourbakhsh et al. (2017) demonstrated that several statistical parameters derived from the SCR, such as the number of SCR peaks, can be used to detect cognitive load. Therefore, we used the frequency of SCR peaks to assess the cognitive load at various phases as participants experienced the tie-dye IVR-SG. The raw EDA data were preprocessed by subtracting a median-smoothed baseline (window size = 8 s) to isolate the phasic component, which was then low-pass filtered with a 5 Hz Butterworth filter to suppress high-frequency noise (Braithwaite et al., 2013). SCR peaks on the phasic signal were identified using the continuous decomposition analysis (CDA) implemented in Ledalab (Benedek and Kaernbach, 2010), with a minimum amplitude threshold of 0.01 μS; responses below this threshold were rejected as artifacts.

#### 4.4.2 Flow short scale and MSLQ-SE

This study utilizes the FSS and the MSLQ-SE subscale to assess participants' flow experience and self-efficacy. The FSS comprises 10 items forming two subscales: fluency of performance (six items; e.g., “My thoughts/activities run fluidly and smoothly”) and absorption by activity (four items; e.g., “I do not notice time passing”), with internal consistencies previously reported as Cronbach's α = 0.93 and α = 0.78, respectively (Engeser and Rheinberg, 2008). An additional three items (11, 12, and 13) measure perceived importance of the task (e.g., “I must not make any mistakes here”; see Appendix A). All items are rated on a seven-point Likert scale with anchors varying from “Not at all” to “Very much.” In the present sample (*N* = 68), Cronbach's α was 0.84 for the fluency subscale and 0.78 for the absorption subscale, both meeting the α ≥ 0.70 threshold commonly recommended for research use.

The MSLQ is widely used to assess students' motivational orientation and learning strategies, and its self-efficacy subscale (MSLQ-SE) captures specific academic self-efficacy (Curione et al., 2019). Nielsen et al. (2017) identified two unidimensional facets within the MSLQ-SE scale: specific academic learning self-efficacy (SAL-SE) and specific academic exam self-efficacy (SAE-SE), each consisting of four items (see Appendix B), with Cronbach's α values reported as 0.87 and 0.89, respectively. The MSLQ-SE items are measured using a five-point Likert scale (1 = not at all, 2 = to a poor degree, 3 = to some degree, 4 = to a large degree, and 5 = perfectly). A higher total score indicates stronger self-efficacy (Duncan and Mckeachie, 1991). Within the current sample, the two subscales demonstrated acceptable internal consistency, with Cronbach's α coefficients of 0.74 (SAL-SE) and 0.75 (SAE-SE), each exceeding the 0.70 benchmark typically considered adequate for research purposes.

#### 4.4.3 Learning assessment

A content knowledge test was developed to assess participants' acquisition of Bai ethnic tie-dye knowledge across three domains: historical and cultural background, artistic characteristics and pattern design, and procedural crafting steps. The pre-test (10 items; maximum = 50 points) captured baseline prior knowledge for use as a covariate, while the post-test (10 different items; maximum = 50 points) measured knowledge acquired through the IVR-SG experience. Both forms covered the same three content domains with a uniform scoring scheme (five points per item), with different items used across tests to avoid testing effects (Johnson and Mayer, 2009). Items were generated based on Bai ethnic tie-dye heritage literature and the instructional content of the IVR-SG, and were reviewed by a panel of three domain experts to ensure content relevance and accuracy (Checa et al., 2023; Makransky and Mayer, 2022). In addition, the IVR-SG automatically recorded procedural performance scores during the practical craft stages, scoring each operation from 1 to 5 based on accuracy and completeness. A weighted composite score was computed across the five stages to yield an overall indicator of procedural task execution within the simulated environment.

To complement these objective indicators and provide triangulatory support for the quantitative findings, an open-ended questionnaire comprising four questions was administered, addressing craft operation experience, perceived difficulty, creative autonomy, and attitudinal change toward the craft (Appendix C). Questions were phrased without reference to any theoretical constructs to elicit unprompted descriptions of the learning experience. Participation in this qualitative component was voluntary, yielding valid responses from 40 participants (natural interaction: 20; motion controller: 20). These illustrative responses were analyzed to examine participants' learning experience through the lens of SDT's three basic psychological needs. Three coders (two authors and one trained research assistant) independently classified each response according to whether its content reflected satisfaction or frustration of autonomy, competence, or relatedness (Ryan and Deci, 2017). A coding rubric was developed to operationalize each construct within the tie-dye IVR-SG context (see Appendix D), and a calibration session was conducted using a subset of responses prior to formal coding. Responses could be assigned to more than one category. Inter-rater agreement was assessed using percent agreement (Grasse et al., 2022), and responses that received votes from two or more coders were included in the final tallies.

## 5 Results

### 5.1 Cognitive engagement

A Linear Mixed Effects Model (LMM) was employed to examine changes in blink rates across different groups during various phases of tie-dye IVR-SG. The dependent variable was blink rate; gender, age, VR interaction method, IVR-SG phase (a five-level factor), and the VR interaction method × IVR-SG phase interaction were entered as fixed effects. Because between-participant variance was negligible, a marginal repeated-measures specification with a heterogeneous within-participant residual structure was used. As shown in Table 3, blink rate differed significantly across IVR-SG phases (*F* = 1275.96, *p* < 0.001) and by gender (*F* = 14.29, *p* < 0.001). The main effect of VR interaction method was not significant (*F* = 0.01, *p* = 0.913), but the VR interaction method × IVR-SG phase interaction was significant (*F* = 49.22, *p* < 0.001), indicating that the influence of interaction method on blink rate was phase-dependent rather than uniform.

**Table 3:** Tests of fixed effects from the linear mixed models for blink rate and EDA peaks.

Effect	Blink rate			EDA peaks		
	*F*	df	*p*	*F*	df	*p*
Gender	14.29	1, 238.6	< 0.001^***^	3.06	1, 243.2	0.082
Age	0.22	1, 238.6	0.636	7.21	1, 243.2	0.008^**^
VR interaction method	0.01	1, 78.3	0.913	0.94	1, 247.4	0.334
IVR-SG phase	1,275.96	4, 114.9	< 0.001^***^	196.28	4, 102.2	< 0.001^***^
VR interaction method × IVR-SG phase	49.22	4, 114.9	< 0.001^***^	14.53	4, 102.2	< 0.001^***^

Denominator degrees of freedom were estimated using Satterthwaite's approximation, yielding effect-specific values. Model fit (model with interaction): blink rate AIC = 1,099.3, BIC = 1,118.2; EDA peaks AIC = 1,036.9, BIC = 1,055.9. ^**^p < 0.01, and ^***^p < 0.001.

EDA metrics were employed as a complementary physiological channel, capturing the autonomic arousal dimension of cognitive load. A parallel model was fitted for EDA peaks (Table 3). EDA peaks differed across IVR-SG phases (*F* = 196.28, *p* < 0.001), and older age was associated with more frequent EDA peaks (*F* = 7.21, *p* = 0.008). Neither the main effect of VR interaction method (*F* = 0.94, *p* = 0.334) nor that of gender (*F* = 3.06, *p* = 0.082) reached significance. As with blink rate, the VR interaction method × IVR-SG phase interaction was significant (*F* = 14.53, *p* < 0.001).

**Figure 7:**
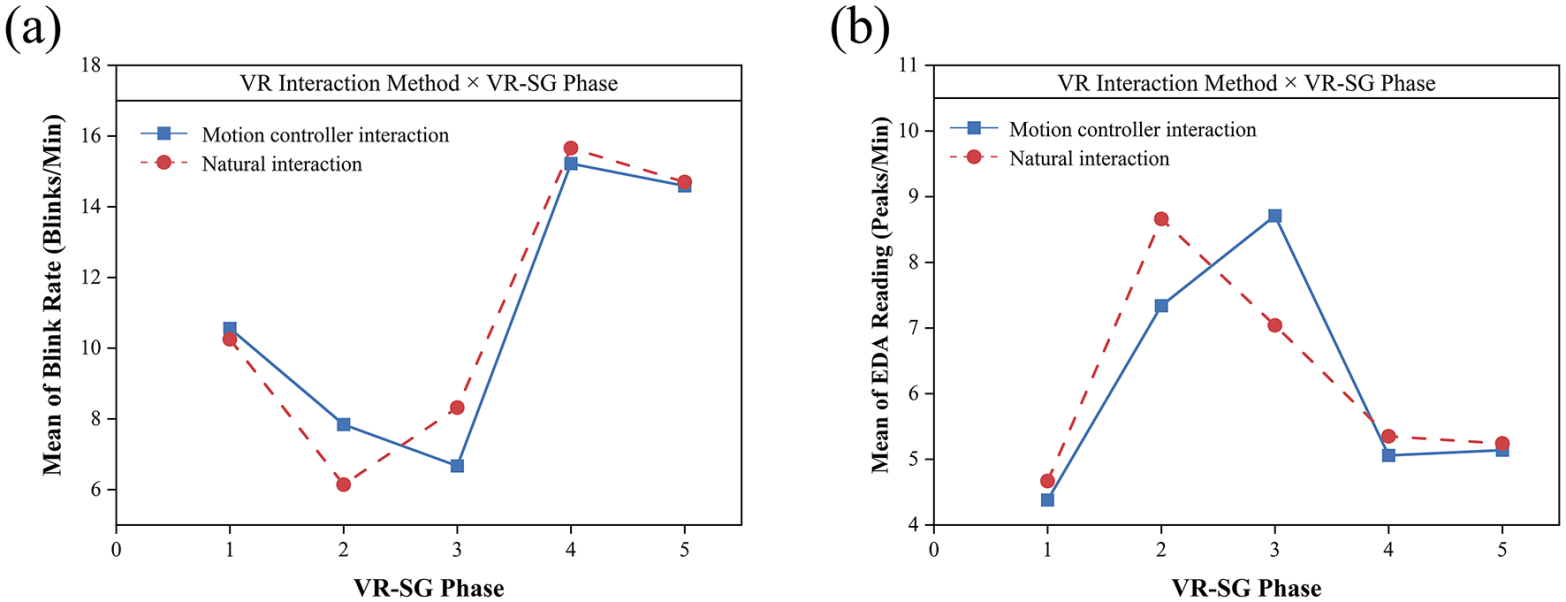
Two-way interactions on **(a)** blink rate and **(b)** EDA reading.

*Post-hoc* analysis indicated that participants in both groups exhibited lower blink rates and more EDA peaks per minute during the Tie-dye Pattern Design phase and the Tie-dye Process Simulation phase compared to other phases. In the Tie-dye Pattern Design phase, participants who used the motion controller interaction had significantly higher blink rates and lower EDA peaks per minute compared to those using natural interaction. Conversely, in the Tie-dye Process Simulation phase, participants using natural interaction showed significantly higher blink rates and lower EDA peaks per minute (see Figure 7 and Table 4).

**Table 4:** Changes in participants' physiological indicators across IVR-SG stages under two VR interaction methods (*N* = 68).

Physiological indicators	IVR-SG phase	Mean (SD)		Independent samples *t*-test^a^	
		Motion controller interaction	Natural interaction	*t*	*p*
Blinks per minute	Tie-dye Digital Museum	10.56 (1.36)	10.25 (0.95)	1.09	0.280
	Tie-dye Pattern Design	7.84 (0.76)	6.14 (0.74)	9.32	< 0.001^***^
	Tie-dyeing Process Simulation	6.67 (0.76)	8.32 (0.66)	−9.56	< 0.001^***^
	Tie-dye Virtual Gallery	15.22 (5.05)	15.66 (6.44)	−0.32	0.753
	Unlock New Resources	14.59 (0.66)	14.70 (0.74)	−0.65	0.516
EDA peaks per minute	Tie-dye Digital Museum	4.38 (0.94)	4.67 (1.18)	−1.11	0.271
	Tie-dye Pattern Design	7.34 (0.77)	8.66 (0.80)	−6.98	< 0.001^***^
	Tie-dyeing Process Simulation	8.71 (1.49)	7.04 (1.55)	4.53	< 0.001^***^
	Tie-dye Virtual Gallery	5.06 (0.75)	5.35 (0.71)	−1.67	0.100
	Unlock New Resources	5.14 (1.64)	5.24 (1.76)	−0.23	0.819

^a^Equal variances observed in F-tests for all variables (*p* > 0.05); ^***^p < 0.001.

**Figure 8:**
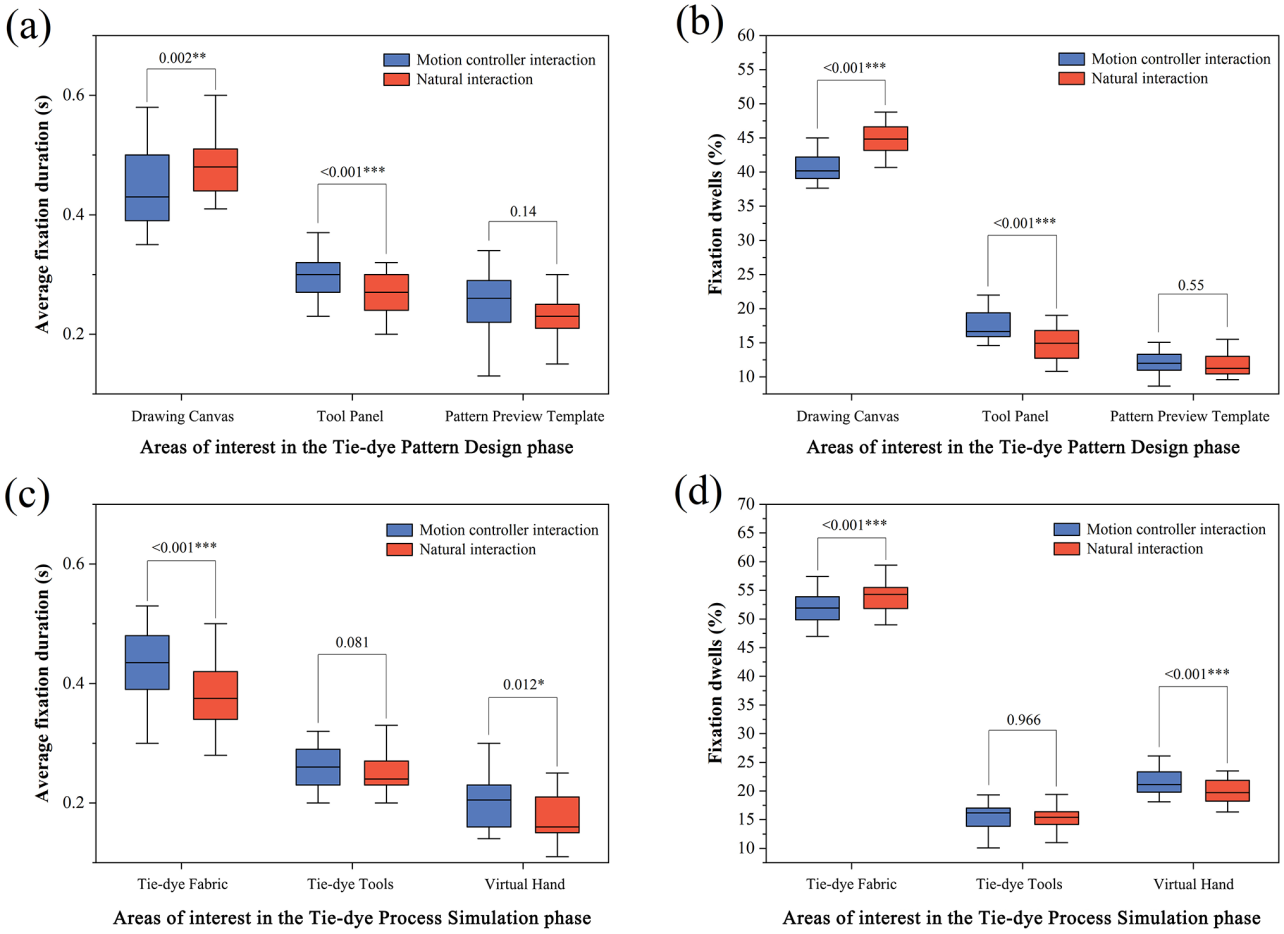
Analysis of user's gaze behavior during the Tie-dye Pattern Design phase and the Tie-dye Process Simulation phase. **(a)** Average fixation duration to three AOIs during the Tie-dye Pattern Design phase. **(b)** Fixation dwells on three AOIs during the Tie-dye Pattern Design phase. **(c)** Average fixation duration to three AOIs during the Tie-dye Process Simulation phase. **(d)** Fixation dwells on three AOIs during the Tie-dye Process Simulation phase. Asterisks exclusively denote statistically significant differences (*p < 0.05, **p < 0.01, and ***p < 0.001). For non-significant comparisons, exact *p*-values are reported directly to ensure full data transparency.

By analyzing participants' fixation behavior within the AOIs during the Tie-dye Pattern Design phase and Tie-dye Process Simulation phase, we investigated the cognitive engagement characteristics of different groups during learning. The results show that both groups initially focused on the tool panel during the Tie-dye Pattern Design stage, followed by attention to the drawing canvas and the pattern preview template (see Table 5). As shown in Figure 8a, during this phase, both groups exhibited longer average fixation durations on the drawing canvas compared to the tool panel, while the pattern preview template received the shortest average fixation duration. Participants using motion controller interaction had a lower average fixation time on the drawing canvas compared to those using natural interaction but higher on the tool panel, with no significant difference on the pattern preview template. Figure 8b illustrates that during the Tie-dye Pattern Design phase, both groups allocated their fixation time across the three AOIs in descending order: the drawing canvas, the tool panel, and the pattern preview template. Motion controller interaction users showed significantly lower fixation dwells on the drawing canvas compared to natural interaction users, while displaying higher fixation dwells on the tool panel. Again, no notable difference was found between the two groups regarding the pattern preview template.

**Table 5:** One-way ANOVA for Time to First Fixation (ms) measures by three different Areas of interest (*N* = 68).

Phase	Interaction method	Areas of interest			*F*	*Post-hoc*
		Drawing Canvas	Tool Panel	Pattern Preview Template		
Tie-dye Pattern Design phase	Motion controller interaction	2.43 (0.28)	0.63 (0.27)	1.57 (0.34)	308.08^***^	Tool panel < Pattern preview template < Drawing canvas
	Natural interaction	2.48 (0.34)	0.45 (0.26)	1.55 (0.25)	437.34^***^	Tool panel < Pattern preview template < Drawing canvas
		Tie-dye Fabric	Virtual Hand	Tie-dye Tools		
Tie-dye Process Simulation phase	Motion controller interaction	0.49 (0.29)	1.47 (0.34)	3.62 (0.43)	677.60^***^	Tie-dye Fabric < Virtual Hand < Tie-dye Tool
	Natural interaction	1.29 (0.29)	0.44 (0.29)	3.61 (0.39)	858.96^***^	Virtual Hand < Tie-dye Fabric < Tie-dye Tool

^***^p < 0.001. Post-hoc pairwise comparisons are reported using the notation A < B < C, where “ < ” indicates a significantly shorter TTFF value. A shorter TTFF corresponds to an earlier fixation onset; thus, the leftmost AOI in each sequence was fixated first (i.e., received the highest visual attention priority).

During the Tie-dye Process Simulation phase, participants using motion controller interaction first fixated on the tie-dye fabric, followed by the virtual hand and then the tie-dye tools. In contrast, participants employing natural interaction initially focused on the virtual hand, subsequently shifting attention to the tie-dye fabric and tie-dye tools (see Table 5). As illustrated in Figure 8c, both groups demonstrated the highest average fixation duration on the tie-dye fabric and the lowest on the virtual hand during this phase. A comparison of the average fixation durations in the same AOI for the two groups shows that participants using natural interaction had a significantly lower average fixation duration on both the tie-dye fabric and the virtual hand compared to those using motion controller interaction. Figure 8d further indicates that all participants allocated the highest fixation dwells to the virtual fabric and the lowest to tie-dye tools. Notably, participants utilizing natural interaction showed a higher fixation dwells on the virtual fabric but a lower fixation dwells on the virtual hand compared to their motion controller interaction.

### 5.2 Subjective learning performance

A *t*-test was conducted to compare the FSS and MSLQ-SE questionnaire results between the two groups, analyzing the impact of different virtual reality interaction methods on participants' flow experience and self-efficacy. As shown in Table 6, participants using natural interaction in the Tie-dye IVR-SG exhibited significantly higher flow experience and self-efficacy compared to those using motion controller interaction.

**Table 6:** The results of *t*-test in subjective learning performance.

Variable	Motion controller interaction	Natural interaction	*t*	*p*
	Mean (SD)	Mean (SD)		
Flow experience	73.82 (6.31)	79.26 (5.17)	−3.89	< 0.001^***^
Self-efficacy	29.88 (1.85)	31.26 (2.53)	−2.57	0.013^*^

^*^p < 0.05, ^***^p < 0.001.

### 5.3 Objective learning results

The pre-test assessed students' baseline knowledge of tie-dye ICH before experiencing the IVR-SG, while the post-test scores reflected their knowledge level after completing the IVR-SG learning. A paired *t*-test comparing pre-test and post-test scores revealed that the post-test scores for the motion controller interaction group were significantly higher than their pre-test scores (*t* = 17.508, *p* < 0.001). Similarly, the natural interaction group also showed a significant improvement in scores (*t* = 23.082, *p* < 0.001). This demonstrates that both interaction methods in the IVR-SG experience are effective for learning tie-dye knowledge. An independent samples *t*-test comparing the pre-test scores between the two groups indicated no significant difference (*t* = 1.582, *p* = 0.118). The non-significant effect between groups is required for a later comparison between group performance (Mayer, 2014). Furthermore, as previously noted, all recruited participants lacked prior experience with immersive VR technologies, thereby ensuring strict baseline equivalence regarding technological familiarity. Prior to conducting the Analysis of Covariance (ANCOVA), preliminary evaluations confirmed that all relevant statistical assumptions were met, including normality, homogeneity of variances, and homogeneity of regression slopes. The analysis of the differences between the two groups was then conducted with an ANCOVA using the pre-test scores as the covariate and the post-test scores as dependent variables. Table 7 summarizes the ANCOVA results, revealing that participants utilizing natural interaction exhibited significantly greater knowledge improvement compared to those employing motion controller interaction (*F* = 10.39, *p* = 0.002). Regarding in-game procedural performance, an independent-samples *t*-test indicated no significant difference in composite scores between the natural interaction group (*M* = 3.46, SD = 0.53) and the motion controller group (*M* = 3.26, SD = 0.42), *t* = 1.778, *p* = 0.080.

**Table 7:** ANCOVA of the post-test for Motion controller interaction and Natural interaction groups.

Variable	Type of learning	N	Mean	*SD*	*F*	*p*
Post-test scores	Motion controller interaction	34	39.38	3.35	10.39	0.002^**^
	Natural interaction	34	41.15	2.90		

^**^p < 0.01.

### 5.4 Correlation and regression analyses

A bivariate intercorrelation analysis was performed to examine the impact of subjective learning performance and cognitive engagement on knowledge gain (Table 8). Based on the theoretical framework established in the literature review (Sections 2.3–2.5), flow experience, self-efficacy, and blink rate were entered as predictors in the regression analysis. The bivariate intercorrelations presented in Table 8 indicated consistent relationships among these variables. As shown in Table 9, Model 1 incorporated participants' demographic characteristics (gender, age, and pre-test knowledge), explaining 14.4% of the variance in post-test knowledge (*F* = 3.59, *p* = 0.018). Pre-test scores had a significant positive impact on post-test scores (β = 0.33, *p* = 0.007), while gender and age had no significant influence on knowledge improvement (*p* > 0.05). Model 2 extended Model 1 by incorporating flow experience, self-efficacy, and blink rate, accounting for an additional 26.1% of variance in post-test knowledge scores. The complete model explained 40.5% of the variance in post-test scores. Specifically, flow experience, self-efficacy, and blink rate all exhibited significant positive effects on post-test knowledge. Based on regression coefficients, the relative influence magnitudes on knowledge acquisition were ranked as follows: pre-test knowledge (strongest), followed by flow experience, self-efficacy, and blink rate. Notably, the observed effect size of the full model (Cohen's *f* ^2^ = 0.68) exceeded the large effect anticipated in the a priori power analysis (Section 4.1), confirming adequate statistical power for the present analysis.

**Table 8:** Bivariate intercorrelations between Tie-dye content knowledge post-test and subjective learning performance, cognitive engagement (*N* = 68).

Variable	Flow experience	Self-efficacy	Blink rate	EDA peaks	Content post-test knowledge
Flow experience	1				
Self-efficacy	0.411^**^	1			
Blink rate	0.152	0.199	1		
EDA peaks	−0.506^**^	−0.437^**^	−0.225	1	
Content post-test knowledge	0.359^**^	0.355^**^	0.306^*^	−0.234	1

^*^p < 0.05, ^**^p < 0.01.

**Table 9:** Regression analysis for variables explaining tie-dye knowledge post-test (*N* = 68).

Variable	Model 1				Model 2			
	β	*SE*	*t*	*p*	β	*SE*	*t*	*p*
Knowledge pre-test	0.325	0.100	2.762	0.007^**^	0.403	0.088	3.592	< 0.001^***^
Gender	0.003	0.777	0.022	0.982	−0.141	0.692	−1.314	0.194
Age	−0.150	0.123	−1.231	0.223	0.038	0.111	0.345	0.731
Flow experience					0.347	0.058	3.045	0.003^**^
Self-efficacy					0.237	0.157	2.122	0.038^*^
Blink rate					0.217	0.246	2.063	0.043^*^
*R* ^2^	0.144				0.405			
*F* for change in *R*^2^	3.594^*^				6.915^***^			
Δ*R*^2^	0.144				0.261			

^*^p < 0.05, ^**^p < 0.01, ^***^p < 0.001.

### 5.5 Qualitative findings

A total of 40 participants submitted valid responses. Inter-rater agreement was high across all coding categories (competence: 96.9%; autonomy: 95.6%; relatedness: 97.5%). Table 10 presents the prevalence of SDT-related themes by interaction condition.

**Table 10:** Prevalence of SDT-related themes by interaction condition.

SDT need	Direction	Natural interaction *n* (%)	Controller Interaction *n* (%)
Competence	Satisfaction	19 (95%)	10 (50%)
	Frustration	9 (45%)	17 (85%)
Autonomy	Satisfaction	17 (85%)	13 (65%)
	Frustration	3 (15%)	12 (60%)
Relatedness	Satisfaction	10 (50%)	3 (15%)
	Frustration	1 (5%)	12 (60%)

Competence-related themes were the most prevalent across both groups. In the natural interaction group, 19 of 20 participants (95%) reported competence satisfaction, predominantly in relation to the Process Simulation phase. Representative responses described the gestural correspondence between virtual and physical craft operations (e.g., “Grab and twist, just like real work. Seeing the patterns form on the fabric felt quite satisfying”). Competence frustration in this group (9 of 20, 45%) was primarily associated with precision demands during the Pattern Design phase (e.g., “Placing small patterns precisely was a bit difficult. My hand trembled slightly in mid-air”). In the motion controller group, competence frustration was more prevalent (17 of 20, 85%) and was commonly linked to button-mapping complexity (e.g., “Had to memorize many button combinations, which key is grasp, which is twist, and had to coordinate timing”), whereas competence satisfaction (10 of 20, 50%) was typically associated with operations involving clear sequential inputs.

Autonomy satisfaction was reported by 17 natural interaction participants (85%) and 13 motion controller participants (65%), with both groups describing comparable creative freedom during the pattern design task (e.g., “I tried several different combination approaches before settling on the final plan”). Autonomy frustration was infrequent in the natural interaction group (3 of 20, 15%) but more common among motion controller participants (12 of 20, 60%), who attributed the restriction not to the range of creative options but to the indirectness of button-mediated interaction (e.g., “Creatively no major restrictions, just the operation was a bit roundabout”). Relatedness was the least frequently coded construct. Relatedness satisfaction was more prevalent in the natural interaction group (10 of 20, 50%) than in the motion controller group (3 of 20, 15%), with responses expressing a desire to share completed works or an interest in the Bai ethnic tie-dye tradition. Relatedness frustration was reported by 1 natural interaction participant (5%) and 12 motion controller participants (60%), with the latter group describing a perceived disconnection between button-based input and the cultural practice.

## 6 Discussion

This study investigates the impact of different VR interaction methods on learning experience and outcomes in VR-based education for handicraft ICH. Through multimodal physiological data analysis, we examined the cognitive engagement of participants in the experimental and control groups during the VR teaching process. Additionally, we assessed flow experience and self-efficacy through validated questionnaires, and knowledge acquisition through a delayed post-test. Below, we discuss our results as they pertain to each of the research questions described in Section 1.

### 6.1 Cognitive engagement

To address RQ1, blink rate and EDA peaks were examined as complementary physiological indicators of cognitive load to evaluate how different interaction methods influenced learners' cognitive load across phases of the IVR-SG. The LMM analysis revealed that the main effect of interaction method was not significant for either indicator, but the interaction method × IVR-SG phase interaction was significant for both blink rate and EDA peaks. *Post-hoc* analysis showed that during the Tie-dye Pattern Design phase, motion controller interaction was associated with higher blink rates and fewer EDA peaks compared to natural interaction, indicating lower cognitive load; the reverse pattern was observed during the Tie-dye Process Simulation phase. The two indicators exhibited concordant response patterns across all conditions, with the modality associated with higher blink rates consistently producing fewer EDA peaks, lending convergent support to the cognitive load interpretation.

By analyzing three visual attention metrics—Time to first fixation, Average fixation duration, and Fixation dwells—we further examined the cognitive process differences among participants using different interaction methods during two phases. During the Tie-dye Pattern Design phase, the motion controller interaction group exhibited significantly lower average fixation duration and fixation dwells in the drawing canvas compared to the natural interaction group. However, the motion controller interaction group showed higher average fixation duration and fixation dwells in the tool panel. This indicates that in virtual reality learning of handicraft ICH, users are more comfortable handling fine and complex operations using motion controller interaction, while natural interactions make it easier to execute simple tasks like command selection. A plausible explanation is that gesture-based interaction recruits larger-scale body movements, which undermines fine motor control during extended precision tasks. In addition, limited gesture-recognition accuracy in complex scenes can elevate error rates. Conversely, when performing selection tasks, gaze-based pointing has been shown to outperform hand-based pointing in speed (Sidenmark and Gellersen, 2019). And this study employs a “gaze selection, hand confirmation” multimodal interaction for tool panel operations, achieving higher accuracy.

Analysis of eye-tracking data during the Tie-dye Process Simulation phase revealed that the first fixation of the motion controller interaction group was on the tie-dye fabric, whereas the natural interaction group initially focused on the virtual hand. This indicates that the natural interaction method gives users a higher sense of presence. The findings of Vuletic et al. (2019) can explain this phenomenon, as natural interaction eliminates the need to map controller button operations to virtual hand movements. Notably, the natural interaction group exhibited shorter average fixation durations on both the tie-dye fabric and virtual hand compared to the motion controller group, indicating that gesture-based interaction facilitates easier comprehension and imitation of craft techniques. Furthermore, the natural interaction group spent more time processing the tie-dye fabric and less time focusing on the virtual hand compared to the motion controller interaction group. This observation is supported by Hoffman (2021), whose research shows that virtual hands can act as attentional distractors. Therefore, using the natural interaction method for virtual crafting operations allows users to maintain higher levels of attention. These phase-dependent differences are consistent with the METUX-based prediction advanced in Section 2.2 and align with an embodied cognition account (Johnson-Glenberg, 2018; Wilson, 2002b): hand tracking provides a direct sensorimotor mapping that frees attentional resources during procedure simulation, whereas precision demands in the Pattern Design phase exceed the control afforded by gross body movements, consuming additional cognitive resources. The qualitative data provide converging evidence for this interpretation. Natural interaction participants who reported competence satisfaction during the Process Simulation phase consistently attributed the fluency of their experience to the correspondence between their hand movements and the virtual craft operations. Those who reported difficulty during the Pattern Design phase linked their frustration to the imprecision of mid-air gesture input for fine-grained selection tasks. Motion controller participants who reported competence satisfaction during pattern design, conversely, emphasized the accuracy and predictability of button-based input. These contrasting accounts complement the physiological findings and indicate that the embodied nature of the interaction modality shapes not only the objective cognitive load but also the subjective experience of competence at the interface level.

### 6.2 Subjective learning performance and Knowledge acquisition

In terms of RQ2 results, we found that compared to the motion controller interaction method, the natural interaction method provides users with a higher flow experience and self-efficacy, as well as better knowledge acquisition. Ruvimova et al. (2020) demonstrated that distractions during IVR experiences can hinder flow states. This finding aligns with our observation, where controller-based participants' attention showed greater allocation to virtual hand representations during the Tie-dye Process Simulation phase, which may partly account for their lower flow experience compared to the natural interaction group. Klingenberg et al. (2024) pointed out that gesture-based commands are better for learning a procedure because the participant physically performs the task. Therefore, we analyzed that in our tie-dye IVR-SG environment, the natural interaction group's operational gestures, guided by system prompts, achieved high consistency with the authentic movements of intangible cultural heritage inheritors in real-world practice. This kinematic alignment may have contributed to participants' higher self-efficacy in learning the procedural steps of this traditional craft within the simulated environment.

The qualitative findings corroborate this account. Natural interaction participants who reported competence satisfaction frequently described a sense of authentic engagement with the craft, whereas motion controller participants more often attributed their confidence to input reliability rather than craft authenticity. Notably, autonomy frustration was considerably more prevalent among motion controller participants (60%) than among natural interaction participants (15%), despite the identical autonomy-related design features across conditions. The coded responses indicate that this frustration stemmed not from the range of creative options, which both groups described as adequate, but from the indirectness of button-mediated interaction in executing creative intentions. A similar pattern was observed for relatedness, with motion controller participants reporting substantially higher relatedness frustration (60% vs. 5%) and describing the experience as procedural knowledge acquisition rather than cultural engagement. These findings suggest that competence frustration at the interface level may cascade into the subjective experience of autonomy and relatedness, consistent with METUX's proposition that need satisfaction across experiential spheres is interconnected (Peters et al., 2018). This cascading effect extends the prediction advanced in Section 2.2 that interaction modality would act most directly on competence, revealing that the motivational consequences of interface-level experience may be broader than a single-need account would suggest.

The post-test knowledge scores of both groups were significantly higher than their pre-test scores, demonstrating that the SDT-guided IVR-SG can effectively teach handicraft ICH knowledge. ANCOVA results revealed that the natural interaction group achieved significantly higher post-test scores than the motion controller group. Despite this advantage in declarative knowledge, the two groups achieved comparable composite procedural performance scores, a dissociation consistent with the scaffolded design of the IVR-SG, in which active and passive scaffolding guided all participants toward successful task completion regardless of interaction modality. The advantage of natural interaction thus appears to operate through deeper knowledge encoding via embodied sensorimotor coupling (Johnson-Glenberg, 2018) rather than through superior immediate task execution. This interpretation is further supported by the contrast with Parong and Mayer (2018), who found no knowledge advantage for natural interaction in a knowledge-oriented VR program; in their context, interactive actions were peripheral to the learning objective, whereas in skill-oriented IVR-SGs such as the present study, the gestural correspondence between virtual and authentic craft movements contributes directly to knowledge encoding, even when immediate procedural performance remains comparable across modalities.

### 6.3 Factors contributing to knowledge acquisition

The regression analysis identified four variables that contributed significantly to post-test knowledge scores: pre-test knowledge, flow experience, self-efficacy, and blink rate. As interaction modality shaped several of these states, they are best understood as mechanisms through which interaction modality may influence learning rather than as effects independent of it. The positive contribution of flow experience, the strongest among the experiential variables, may reflect the heightened concentration and engagement afforded by natural interaction (Madden et al., 2020). Self-efficacy similarly contributed to knowledge acquisition, consistent with the broader finding that confident learners tend to persist longer and engage more deeply with learning tasks (Seijts and Latham, 2011). Among the physiological indicators, blink rate contributed positively while EDA peaks did not enter the model as a significant predictor. As established in Section 2.5, blink rate indexes the perceptual-attentional demand of visual information processing, whereas EDA indexes autonomic arousal. The selective contribution of blink rate suggests that in the present visually guided craft-learning context, the perceptual-attentional dimension of cognitive load is more closely associated with knowledge encoding than the autonomic arousal dimension: participants who experienced lower perceptual-attentional demand were better positioned to attend to instructional details and assimilate procedural knowledge (Huang et al., 2010). The weak bivariate correlation between blink rate and EDA peaks corroborates this two-channel interpretation (Ayres et al., 2021; Nilsson et al., 2022), while the concordant condition-level response patterns of both channels across learning phases (Table 4) support their complementary use in characterizing cognitive load.

Beyond specific ICH applications, these findings provide a theoretical heuristic for educational technology design by explicitly outlining a three-tiered causal chain for immersive learning. First, the interaction modality serves as the root independent factor governing interface-level cognitive load and attention allocation. Second, the kinematic alignment of this modality with real-world tasks directly shapes the learners' experiential states, which are empirically measured here as flow and self-efficacy, without requiring an abstract proxy for immersion. Finally, the broader SDT motivational mechanisms act as the translation engine, wherein the fulfillment of these psychologically embodied needs ultimately converts into declarative knowledge retention. From a practical design perspective, future research should address camera occlusion and enhance motion recognition accuracy to further reduce cognitive load during precision operations.

### 6.4 Limitations and future study

Methodologically, our approach to data capture presents certain analytical boundaries. While self-efficacy and flow served as proximal indicators of competence and autonomy, future research should incorporate validated instruments such as the Basic Psychological Need Satisfaction scale (Chen et al., 2015) or the METUX TENS scales (Peters et al., 2018) to directly test these motivational pathways. This should be complemented by phase-specific flow measurements (Pearce et al., 2005) and granular logging of scaffold engagement, which would unpack the real-time interaction dynamics currently obscured by overall retrospective scores. Furthermore, while our open-ended questionnaire provided valuable triangulatory support, its voluntary 59% response rate signals a potential self-selection bias toward more highly motivated participants.

On an operational level, the physical constraints of VR hardware and experimental setting warrant consideration. Because the two interaction conditions relied on distinct hardware configurations, true double-blinding was unfeasible; although experimenter involvement was strictly restricted, demand characteristics cannot be completely dismissed, and future studies could adopt a crossover design in which each participant experiences both interaction modalities to reduce such expectancy effects. Additionally, while participants completed the tasks without reported discomfort, future work involving extended motor tasks would benefit from multidimensional physical and visual fatigue tracking (Fan et al., 2023). Finally, relying on a VR-naïve, homogenous university student sample limits immediate generalizability. Validating these interaction synergy mechanisms across younger school cohorts, non-academic working professionals, or diverse cultural heritage traditions remains a vital frontier for future ICH education research.

## 7 Conclusions

To study the impact of different VR interaction methods on virtual reality education for handicraft ICH, this research utilizes SDT to design an IVR-SG for teaching the ICH of Bai ethnic group's tie-dyeing in China. The game consists of five stages of learning activities, each accompanied by specific tasks, learning objectives, and interaction behaviors. The combination of multichannel objectives (eye tracking, EDA) and subjective data (self-reports) provides a multimodal approach by which important continuous data about learning processes can be captured. The LMM analysis of participants' cognitive load indicates that in the VR teaching of handicraft ICH, using a controller interaction method is more advantageous for completing precise operations, while using natural interaction makes it easier to simulate craft processes. Further analysis of users' eye movement behavior reveals that during the handicraft production process simulation, traditional controller interactions were associated with greater attention allocation to the virtual hand. In contrast, natural interaction allows users to maintain better focus on the operation target and execute command selections more swiftly. Results on flow experience and self-efficacy demonstrated that natural interaction provided a more immersive VR experience, enhancing learners' confidence in performing the craft procedures within the simulated environment. Additionally, the knowledge test scores of both participant groups demonstrate that natural interaction can result in better knowledge acquisition, while both groups achieved comparable levels of in-game procedural performance. Regression analysis further indicated that pre-test knowledge, flow experience, self-efficacy, and blink rate contributed significantly to knowledge acquisition. Supplementary qualitative analysis further revealed that these interaction-modality effects were reflected in participants' reported experience, and that competence frustration at the interface level may cascade into the subjective experience of autonomy and relatedness, extending the scope of the METUX-based theoretical predictions.

These findings highlight the significant potential of natural interaction methods in VR education for intangible cultural heritage (ICH) craftsmanship, as demonstrated by the Bai ethnic tie-dye case study. Beyond pedagogical effectiveness, the IVR-SG developed in this research offers a scalable pathway for disseminating handicraft ICH beyond its geographical origins, thereby raising broader public awareness and appreciation in the digital age. Future research should extend this framework to other handicrafts with distinct motor demands, diverse learner populations, and broader cultural contexts. The transfer of in-game procedural learning to actual hands-on craft performance also warrants investigation. Additionally, refining the precision of natural interactions and exploring novel modalities will further enhance learning experiences and outcomes, ultimately empowering VR technology to better facilitate the preservation and evolution of ICH.

## Data Availability

The raw data supporting the conclusions of this article will be made available by the authors, without undue reservation.
