# Supplementary material for: Enhancing handicraft intangible cultural heritage learning through immersive VR serious games: a multimodal study on natural interaction effectiveness
Source: Front Psychol. 2026 Jul 16;17:1880795. doi: 10.3389/fpsyg.2026.1880795 (PMC13422570; doi:10.3389/fpsyg.2026.1880795)
Supplement: Supplementary file 1 [file Supplementary_file_1.docx]

**Abbreviations**

The following abbreviations are used in this manuscript:

| **Abbreviation** | **Full Term** |
| --- | --- |
| AFD | Average Fixation Duration |
| AOI | Area of Interest |
| EDA | Electrodermal Activity |
| FSS | Flow Short Scale |
| GSR | Galvanic Skin Response |
| HMD | Head-Mounted Display |
| ICH | Intangible Cultural Heritage |
| IVR | Immersive Virtual Reality |
| LMM | Linear Mixed Model |
| MSLQ | Motivated Strategies for Learning Questionnaire |
| SAE-SE | Specific Academic Exam Self-Efficacy |
| SAL-SE | Specific Academic Learning Self-Efficacy |
| SCR | Skin Conductance Response |
| SDT | Self-Determination Theory |
| SG | Serious Game |
| TTFF | Time to First Fixation |
| VR | Virtual Reality |

**Appendix A**

**Table A1.** Flow short scale (FSS).

| **NO.** | **Item** |
| --- | --- |
| 1 | I feel just the right amount of challenge |
| 2 | My thoughts/activities run fluidly and smoothly |
| 3 | I do not notice time passing |
| 4 | I have no difficulty concentrating |
| 5 | My mind is completely clear |
| 6 | I am totally absorbed in what I am doing |
| 7 | The right thoughts/movements occur of their own accord |
| 8 | I know what I have to do each step of the way |
| 9 | I feel that I have everything under control |
| 10 | I am completely lost in thought |
| 11 | Something important to me is at stake here |
| 12 | I must not make any mistakes here |
| 13 | I am worried about failing |

Note. Fluency of performance: 2, 4, 5, 7, 8, 9. Absorption by activity: 1, 3, 6, 10. Perceived importance or outcome importance: 11, 12, 13.

**Appendix B**

**Table B1.** MSLQ-SE.

| **NO.** | **Item** |
| --- | --- |
| 1 | I believe I will receive an excellent grade in this class. (SAE-SE) |
| 2 | I'm confident I can do an excellent job on the assignments and tests in this course. (SAE-SE) |
| 3 | I expect to do well in this class. (SAE-SE) |
| 4 | Considering the difficulty of this course, the teacher, and my skills, I think I will do well in this class. (SAE-SE) |
| 5 | I'm certain I can understand the most difficult material presented in the readings for this course. (SAL-SE) |
| 6 | I'm confident I can understand the basic concepts taught in this course. (SAL-SE) |
| 7 | I'm confident I can understand the most complex material presented by the instructor in this course. (SAL-SE) |
| 8 | I'm certain I can master the skills being taught in this class. (SAL-SE) |

**Appendix C**

Open-Ended Questionnaire

| **Item** | **Topic** | **Question** |
| --- | --- | --- |
| **Q1** | Craft operation experience | During the Tie-dye Process Simulation phase (tying, dyeing, washing, drying), which operation did you find most smooth or gave you the greatest sense of accomplishment? Please describe the specific situation. |
| **Q2** | Perceived difficulty | Was there any operation that you found difficult or needed repeated attempts? Please describe the specific situation and what made it difficult. |
| **Q3** | Creative autonomy | During the pattern design phase, to what extent did you feel you could create according to your own ideas? Was there any moment when you felt restricted? |
| **Q4** | Attitudinal change | After this experience, how has your understanding of and confidence in the tie-dye craft changed? Would you want to share your work with others or continue learning about this craft? |

**Appendix D**

SDT-Based Coding Rubric

| **Construct** | **Definition** | **Example Indicator** |
| --- | --- | --- |
| **Competence Satisfaction** | Participant describes feeling effective, capable, or accomplished during a craft operation, or reports that the interaction felt smooth, natural, or easy to control. | *"The gestures for the tying step felt very natural, I could do it smoothly without thinking about the controls."* |
| **Competence Frustration** | Participant describes difficulty, repeated errors, inability to achieve intended actions, or frustration with interaction accuracy. | *"I kept having to repeat the pinch gesture because the system did not recognise it properly."* |
| **Autonomy Satisfaction** | Participant describes feeling free to choose, create, or operate according to own preferences and ideas. | *"I liked that I could design my own pattern and choose whichever motifs I wanted."* |
| **Autonomy Frustration** | Participant describes feeling restricted, forced to follow a fixed sequence, or lacking meaningful choice. | *"I felt like I had to follow the exact steps and could not explore on my own."* |
| **Relatedness Satisfaction** | Participant describes connection to the craft tradition, desire to share work with others, or cultural appreciation. | *"After the experience I want to show my friends what I made, it feels like real tie-dye."* |
| **Relatedness Frustration** | Participant describes disconnection from the craft, lack of cultural resonance, or absence of social meaning. | *"I did not really feel connected to the cultural background of the craft."* |
